# Notes and News

**Published:** 1892-04-02

**Authors:** 


### Notes and News.

Collected and Communicated.

At the annual meeting of University College Hospital, the Chairman alluded to the contemplated rebuilding of the hospital. He remarked that some years ago they might have had a magnificent site, having the University College on the one side and the Euston Road on the other. They would in that case have secured a hospital free from noise and turmoil. He did not know whether Parliament would interfere to enable them to purchase such site at present. There was the possibility of obtaining the whole of the college buildings. They would then have a much larger site; the present one being somewhat small. The hospital is a most useful one, and we regret to see that the debt of £14,000 still remains to be cleared off, whilst donations during the year have fallen short of those previously received. Nevertheless, the year has been one of great activity, owing to the epidemic of influenza, and other causes. The hospital has lost one or two good friends during the year. In memory of one, Dr. Cyril Jecks, resident medical officer, a bed has been endowed and named.

A remarkable article on the formation of the skull in relation to crime, and especially to the crime of murder, appears in the *Globe*. In it we are told that the study of the formation of the skulls of murderers has been diligently pursued by scientists until they are now able to make some remarkable disclosures. They say that atavism, that is the reversion of type in the evolutionary series, is almost certain to produce the criminal, and in the case of one criminal, who had an apparently insatiable thirst for murder, the skull was of a lower type than that of a monkey. The brain, it is said, was so depressed in the fore part, as to render the man devoid of all intellectual qualities. It is stated that almost without exception, wilful, deliberate murderers are possessed of heads of such apparently peculiar formation, that they would at once be stamped as dangerous and vicious men by any one familiar with the subject of skull formations. These instances, and many similar ones in the same article, must offer subjects for the reflection of those interested in the doctrine of responsibility.

The Hospital for Epilepsy and Paralysis, Regent's Park, has just held its annual meeting. Mr. E. Boulnois, M.P., presided, and in his address showed that he fully appreciated the immense benefit such a charity confers upon a class above all dependent on the help of others. He highly recommended the system of payment by patients according to means, a system which has been adopted by nearly all other countries, and which must be resorted to in England sooner or later. The plan has in no wise interfered with either the usefulness nor popularity of this hospital, as is proved by the pressing need for new buildings, for which purpose Mr. Pearce Gould, Surgeon to the institution, informs the public that the sum of £20,000 will be necessary.

The London Sanitary Protection Association present a report this year which will prove satisfactory to those desirous of sanitary advance. Four hundred and seventy-five tenements received inspection for the first time, which brings the total of inspections during the decade to 4,591. The inspection insisted upon is of the most complete and searching character, and the water test is always applied. The necessity of such inspection is revealed by the report. Of the 475 houses inspected, 228, or 48 per cent., were pronounced very bad, and only 36 were found to be very good. If we are to believe common report, a complete inspection of London houses would show a still worse aspect. The dwellings of the poor have not as yet come under the action of the Association; but its desire to assist forward a work so truly essential, has resulted in a donation of £10 by the Council to the "Mansion House Council on the Dwellings of the Poor," who are doing similar work to that of the Association. During the ten years of its existence the society has increased its members from 192 to 1,613—an excellent proof of popularity. The charges for membership and inspection are only such as cover the working expenses.

The annual meeting of St. Mary's Hospital was marked by several important announcements. The new wing of the hospital is to be called the Clarence Memorial Wing, and of this the foundation-stone is to be laid in October by their Royal Highnesses the Prince and Princess of Wales. Prince George has accepted the presidency of the hospital, and the Duke of Cambridge will preside at the annual dinner. These facts speak for themselves as to the interest that is growing in this enterprising hospital. The proposed additions to the hospital will entail the expenditure of a large sum of money, but the advantage to all classes of the community will be undeniable. The Dean points out in his report that the lying-in wards alone will be an immense acquisition, both to occupants and students, enabling the latter, as they will, to carry out their instruction under direct guidance from eminent authorities. Any advantages in the instruction of future medical practitioners is a direct gain to the richer classes, who should come forward with willing assistance. The hospital is sending out neat little books for the collection of specified sums, and we believe that anyone applying for the same would not have much trouble in turning the pages into gold for the furtherance of the extension scheme.

In a previous issue we gave the result of the competition for the planning of the Birmingham New General Infirmary. Some of our readers may be interested to learn a few particulars of the scheme which Mr. Henman proposes to carry out. There is one entrance for both out and in patients, the latter reaching the interior of the hospital through a separate corridor. The blocks form a quadrangle. A main corridor runs through each. In the administrative block are wards running right and left. The laundry and pathological department occupies a block, with wards on each side. A septic ward block, with gynæcological ward, is also a separate building, as is the nurses' home. The waiting-hall is intended to be used for purposes of recreation also. It is surrounded by a ring of top-lighted retiring-rooms, in connection with consulting-rooms, with lavatory accommodation. On the opposite side of the waiting-room a corridor leads to a twenty-four-bed male surgical ward. The ward is rectangular in shape, with canted corners. The baths, closets, &c., are placed in angle turrets at the north end. A kitchen and two single-bed wards are placed at the other end of the ward. The two children's wards are circular. The corridors are well ventilated, and the main corridor opens out into a conservatory. The staircases are closed in to avoid draughts. There seem, in fact, to be few details of arrangement into which Mr. Henman has not carefully looked.

There is no doubt but that this is the age of advertisement. And advertisement plays an important part not only in our business, but in our charities also. The first point in any case is to attract the eye. The eye being attracted, the more directly the requirement of the case is put forward in the case of charity appeals the better it is. The public are open to being convinced, and when convinced are often willing enough to give. But we must remember, firstly, that the public does not like trouble; and, secondly, that "many men mean many minds," and the paths of approach must be various. We have before us a number of appeals from hospitals, and it is interesting to note how the compilers bring their wants forward. There is no doubt that Lord Sandhurst lends very effective help to the Middlesex Hospital by his direct little circular, and the pamphlet accompanying it is admirably calculated to appeal to all sorts and conditions of men. The illustrations are of many interests. Mothers' hearts will warm towards the little one in its cot, and the kind nurse occupied in amusing it. Admiration will be excited by the ingenious method in which a typhoid patient is being bathed, whilst those confined within the four walls of a sick room will look with envy on the delightful *al fresco* picture in the grounds of the hospital. This little pamphlet is, perhaps, the most complete advertisement of the kind that we know. Nevertheless, we cannot but admire the directness of the appeal attached to the report from the Hospital for Sick Children, at Newcastle-on-Tyne. "Wanted, £500 additional subscriptions," stares at us in clear, red letters, and we cannot overlook them. This is hitting fair from the shoulder, and we cannot doubt that those who have the wherewithal to give, will immediately respond, to the benefit of the hospital. Much depends on the way of asking, and we recommend the consideration of those appealing that one effective hit is worth a number of small blows which may pass unnoticed.

